# Unusual Presentation of Cystic Papillary Thyroid Carcinoma

**DOI:** 10.1155/2012/732715

**Published:** 2012-10-24

**Authors:** Vijayraj S. Patil, Abhishek Vijayakumar, Neelamma Natikar

**Affiliations:** Department of General Surgery, Victoria Hospital, Bangalore Medical College and Research Institute, Bangalore 560002, India

## Abstract

Papillary thyroid carcinoma is the most common thyroid malignancy, accounting for 80% of all thyroid cancers. The most common presentation of thyroid cancer is an asymptomatic thyroid mass or a nodule. Usually as thyroid enlarges, it extends in to mediastinum. Papillary thyroid carcinoma presentation as multiple true cystic swelling extending from neck to anterior chest wall in subcutaneous plane is not present in the literature. We present a rare case of cystic papillary thyroid carcinoma which is presented as subcutaneous swelling with sinus formation.

## 1. Introduction

Papillary thyroid carcinoma is the most common thyroid malignancy, accounting for 80% of all thyroid cancers [1]. The most common clinical presentation is a solitary thyroid nodule. A pure cystic nodule, although rare (<2% of all nodules), is highly unlikely to be malignant [2]. 

 Cystic neck masses appearing in the anterior or posterior triangles of the neck are usually benign. However, they may occasionally have a sinister origin and should be investigated rigorously [3]. Solitary cervical cystic mass is an uncommon presentation of papillary thyroid carcinoma (PTC). Usually as thyroid enlarges, it extends in to mediastinum due to its anatomic location under investing layer of the deep cervical fascia. 

We report a case of papillary thyroid carcinoma presented as a multiple true cystic swelling in neck extending over the chest in subcutaneous plane with a discharging sinus a very unusual presentation. Papillary thyroid carcinoma presented as multiple true cystic swelling extending from neck to breast is not present in literature. The case was diagnosed after surgery by histopathological examination. 

## 2. Case Report

**Figure 1:**
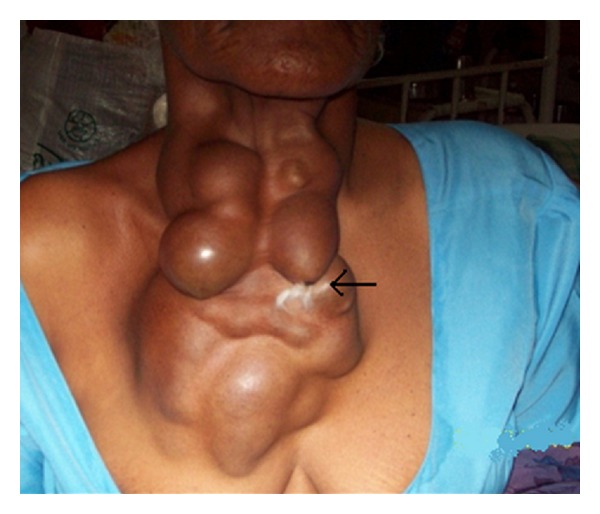
Preoperative photo multiple cysts in neck and chest with sinus (arrow).

A 60-year-old female patient was presented with swelling in front of the neck since 12 yrs, initially small in size started in thyroid region and gradually increased to present size (25 × 15 cm) with watery discharge form swelling of 6 months duration (Figure 1).

**Figure 2:**
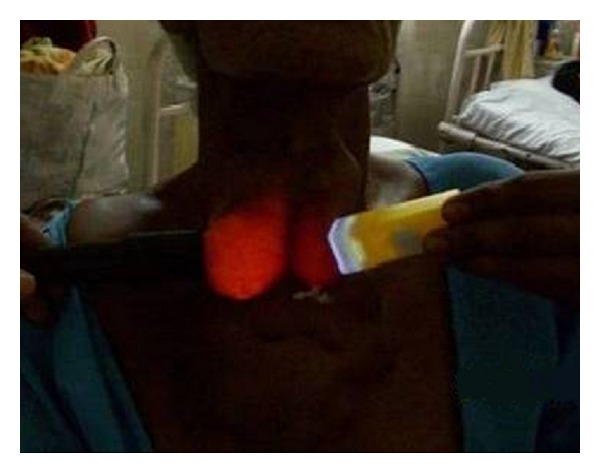
Transillumination of swelling.

**Figure 3:**
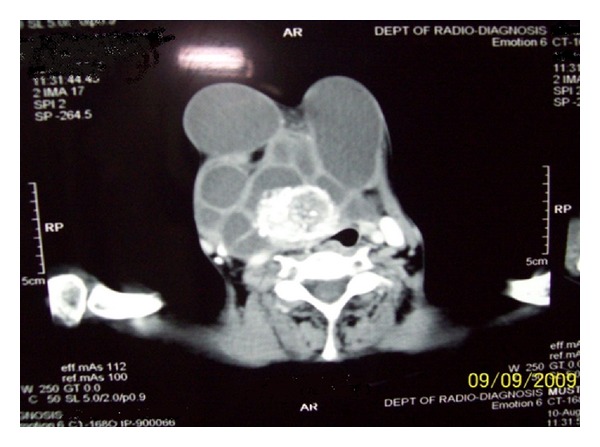
Computer tomaographic image showing multiple cysts arising from right lobe of thyroid.

**Figure 4:**
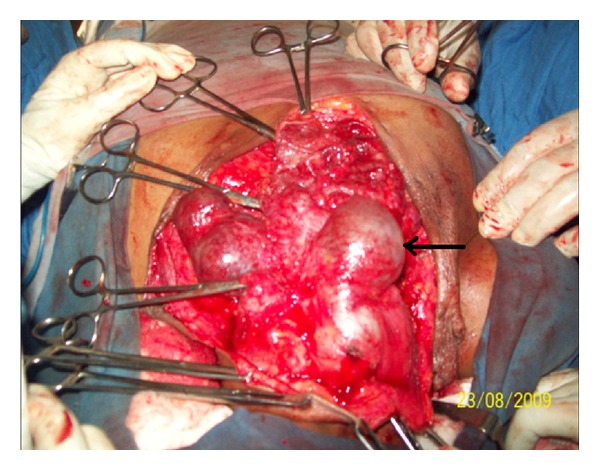
Intraoperative photo showing multiple cysts.

**Figure 5:**
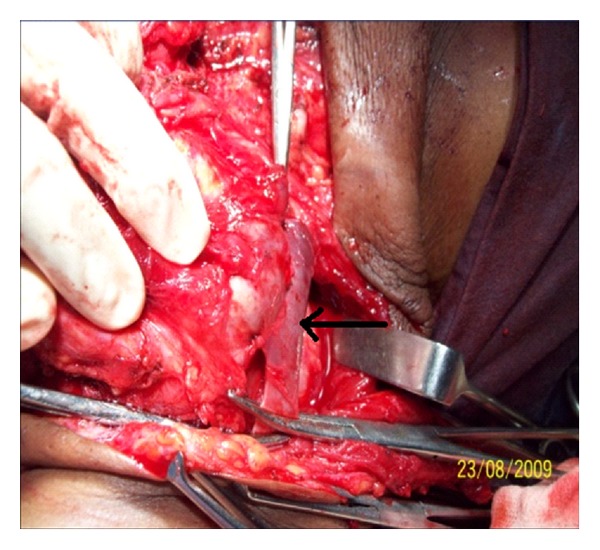
Cyst adherent to Internal jugular vein (arrow).

**Figure 6:**
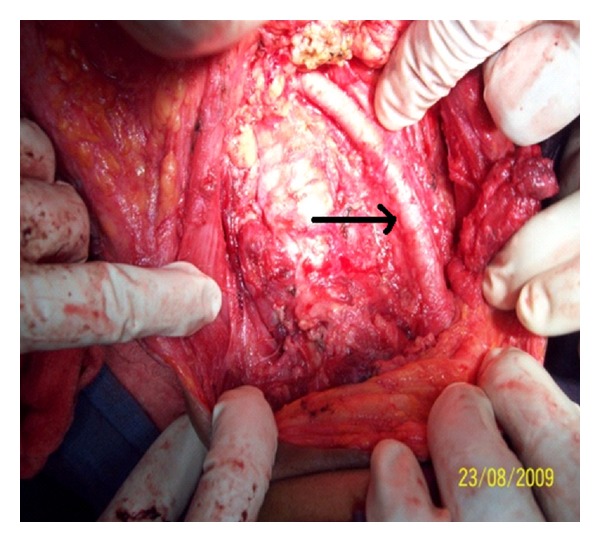
Picture showing right carotid artery (arrow) and trachea on removal of cysts and total thyroidectomy.

**Figure 7:**
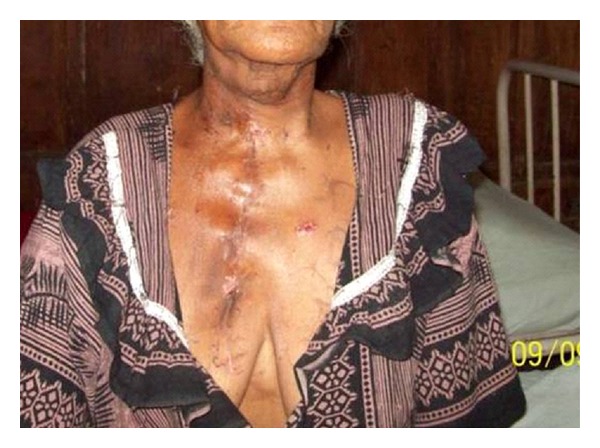
Postoperative photo.

On examination there were multiple cystic swellings extending from thyroid cartilage to mid sternum. There were about 12–15 cysts with features of intercommunication. The whole swelling moved with deglutition and there was no independent mobility of cysts. A sinus was present on right side of neck with active serous discharge (Figure 2). Cervical lymph nodes were not enlarged. On indirect laryngoscopy, no abnormality was detected. Thyroid profile was within normal limits. Neck and chest X-ray showed increased soft tissue density seen in neck region extending up to upper sternal region and indenting trachea. Fine Needle Aspiration Cytology of the swelling showed features suggestive of nodular goitre. Ultrasound of the neck showed multiple anechoic areas with areas of calcification and internal septae in the neck extending from carotid level to midsternal level. Lesion seemed to be arising from thyroid tissue showing minimal vascularity suggestive of multicystic goitre or lymphangioma. On computer tomography there were multiple fluid attenuation coalescent cystic lesions in subcutaneous plane of neck and proximal thorax. There were no inhomogenous enhancing areas within the cyst. Thyroid gland could not be visualized seperately from cysts. There was no infiltration of surrounding structures or lymphadenopathy. The cystic lesions seem to be arising from thyroid gland and only part of left lobe of thyroid was visible (Figure 3). Patient was subjected for surgery. Under general anesthesia Kocher's horizontal incision taken over the swelling and in middle incision extended vertically downwards over right breast as T incision and flaps raised. Fluid in cyst was clear light brown color. Some cysts were intercommunicated and cysts were found arising from right lobe and isthmus of thyroid (Figure 4). On the right side cysts were adherent to internal jugular vein [IJV] (Figure 5) and common carotid artery (Figure 6). Internal jugular vein was ligated and divided. Cysts were separated from right carotid artery. Total thyroidectomy done along with excision of all cysts and skin around sinus. Postoperative period was uneventful (Figure 7). On histopathological examination of specimen papillary carcinoma of thyroid was found and sinus tract was free of tumor cells. Patient underwent an radioactive iodine ablative postoperatively and is on suppressive dose of thyroxine since then.

## 3. Discussion

The most common benign cervical cysts are branchial cleft cysts, dermoid cysts, teratoma, epidermoid cysts, and cystic hygromas. Due to the increasing incidence of oropharyngeal carcinoma, cystic masses of the neck can also be metastases from an oropharyngeal or tonsillar tumour [4]. Rarely, PTC is presented as a cystic neck mass without palpable lesion in the thyroid gland [5]. The origin of these cysts is controversial. Some authors think that it represents a malignant transformation of ectopic thyroid tissue others believe they represent a secondary metastatic spread from occult thyroid lesion to the lymph node which underwent central liquefaction with cystic formation [6]. 

Papillary thyroid carcinoma is a slowly growing neoplasm which explains the relatively long duration in our patient. The long duration of such cysts in young aged patients can lead to incorrect provisional diagnosis of benign cysts [7]. Papillary thyroid carcinoma with extrathyroidal extension (ETE) occurs in 4% to 16% of cases and carries with it an increased risk of disease recurrence and death [8]. Common ETEs include involvement of recurrent laryngeal nerve, larynx, trachea, and esophagus. Involvement of skin with sinus formation is rare, only few cases are reported [9]. 

High-resolution sonography is widely used to detect and characterize thyroid nodules. Sonographic features that are found to be associated with an increased risk of malignancy include a predominantly solid composition, hypoechogenicity, absence of a hypoechoic halo, presence of microcalcifications, irregular margins, and intranodular vascularity [10]. On sonography, papillary thyroid carcinoma appears most commonly as hypoechoic solid lesions with a smooth or irregular contour, intrinsic vascularity, and lacks a hypoechoic halo. Also in a study on papillary thyroid carcinoma by Jun et al. [11], it was shown that only 5% of lesions were purely cystic and that 69% of all papillary thyroid carcinomas had marked intrinsic hypervascularity: flow in the central part of the tumor that was greater than that of the surrounding parenchyma; 22% had perinodal vascularity. Chan et al. [12] reported that all predominantly cystic papillary thyroid carcinomas in their study had some intrinsic blood flow. Lymph node metastases are common in patients with papillary thyroid carcinoma, and approximately 50% of patients have cervical lymph node metastases at the time of their initial presentation [13]. In up to 20% of all patients, lymph node metastases may even be the sole or initial manifestation of disease (occult primary tumor) [14]. Approximately 40% (reported range, 21–50%) of all lymph node metastases from papillary thyroid carcinomas have the tendency to completely cavitate a lymph node by cystic degeneration and thus may mimic an apparently benign cervical cyst [15]. 

Sonographic features of cystic papillary thyroid carcinoma or metastatic cystic nodes include multiple or solitary cyst with internal septation, internal nodules, and punctuate calcification. Histologically, the internal septations were either caused by solid, papillary structures on fibrovascular stalks, by connective tissue filaments, or by the remaining streaks of lymphoid tissue (residual after the liquefaction necrosis or accompanying intranodal hemorrhage). The papillary structures on fibrovascular stalks are especially known to be typical for papillary thyroid carcinomas and are associated with psammoma bodies and paucity of colloid in the primary tumors [16]. The sonographic visible internal nodules were either caused by partly organized colloid or thyroid tumor cells forming follicles with subsequent colloid production, by (partly) organized intranodal hemorrhage and debris, or by clusters of remaining lymphoid tissue surrounded by carcinomatous elements.

According to consensus guidelines on the management of thyroid nodules detected on sonography, with a specific focus on which nodule should undergo sonographically guided FNA by Society of Radiologists in Ultrasound (SRU) [10]. Predominantly cystic nodules (>75% cystic) without microcalcifications have a very low likelihood of malignancy. Similarly, lack of internal flow on color Doppler imaging suggests a lower likelihood of malignancy. As a result, the consensus statement did not recommend FNA for cystic or almost completely cystic nodules [10]. FNAC is less sensitive in the diagnosis of cystic neck masses compared with solid masses having a false negative rate ranging from 50% to 67% [17]. In a study by Muller et al. [18], FNAC had false negative rate of 45% in diagnosing cystic papillary thyroid carcinoma. This high level of false negativity is due to sampling error and not cytological misdiagnosis. Ultrasound guided FNAC that can obtain material from the wall and solid part of the cyst increases the accuracy of FNAC [19].

Wunderbaldinger et al. [20] showed 80% of cystic nodes in papillary thyroid carcinoma the aspirated fluid as brownish viscous and had elevated thyroglobulin levels. Som et al. [21] and King et al. [22] who hypothesized that high intranodal concentration of thyroglobulin reflect areas of high attenuation on CT and inhomogeneous intracystic signal changes on MR imaging, respectively. Therefore, the fluid from these cystic lesions should be sent for thyroglobulin analysis whenever an unclear cystic mass in the neck is present or when the diagnosis of such a mass is in question.

Excisional biopsy is essential to detect papillary thyroid carcinoma in patients with negative FNAC. If clinical or radiological suspicion of malignancy is present, frozen section analysis of the specimen may help to proceed for a total thyroidectomy with modified radical neck dissection at the same setting.

Adequate surgery by removing the cyst, all of the thyroid tissue and accessible involved lymph nodes is essential for better prognosis. Postoperative radioiodine ablation of thyroid remnant with suppressive thyroxine dose is advocated to reduce recurrences. Followup with serial thyroglobulin estimation can help early recurrences or metastasis. 

Papillary thyroid carcinoma (PTC) clinically behaves in an indolent fashion and carries an excellent prognosis >90% survival at 20 years. ETE has a 54% 15 year survival and 29% 30 year survival [8].

## 4. Conclusion

Papillary thyroid carcinoma is the most common of thyroid malignancies. Though most present with solitary thyroid nodule or as lymph node mass. Unusual presentation like solitary cystic nodal mass or multiple cystic mass in neck must be considered. Rarely can it present with sinus formation in long standing cases. Ultrasonography can help in differentiating benign from malignant cystic lesions of neck. Though FNAC is not very accurate in diagnosis of cystic lesions. Aspirated fluid thyroglobulin and thyroid transcription factor levels may help to differentiate cystic thyroid carcinomas from benign cystic lesions. Complete cyst excision with total thyroidectomy and node dissection provides good prognosis even in setting of extrathyroidal extension.
